# System biology analysis of long-term effect and mechanism of Bufei Yishen on COPD revealed by system pharmacology and 3-omics profiling

**DOI:** 10.1038/srep25492

**Published:** 2016-05-05

**Authors:** Jiansheng Li, Peng Zhao, Liping Yang, Ya Li, Yange Tian, Suyun Li

**Affiliations:** 1 Henan University of Traditional Chinese Medicine, Zhengzhou 450046, China; 2 Collaborative Innovation Center for Respiratory Disease Diagnosis and Treatment & Chinese Medicine Development of Henan Province, Zhengzhou 450046, China; 3 Department of Respiratory Diseases, the First Affiliated Hospital of Henan University of Traditional Chinese Medicine, Zhengzhou 450000, China

## Abstract

System pharmacology identified 195 potential targets of Bufei Yishen formula (BYF), and BYF was proven to have a short-term therapeutic effect on chronic obstructive pulmonary disease (COPD) rats previously. However, the long-term effect and mechanism of BYF on COPD is still unclear. Herein, we explored its long-term effect and underlying mechanism at system level. We administered BYF to COPD rats from week 9 to 20, and found that BYF could prevent COPD by inhibiting the inflammatory cytokines expression, protease-antiprotease imbalance and collagen deposition on week 32. Then, using transcriptomics, proteomics and metabolomics analysis, we identified significant regulated genes, proteins and metabolites in lung tissues of COPD and BYF-treated rats, which could be mainly attributed to oxidoreductase-antioxidant activity, focal adhesion, tight junction or lipid metabolism. Finally, based on the comprehensive analysis of system pharmacology target, transcript, protein and metabolite data sets, we found a number of genes, proteins, metabolites regulated in BYF-treated rats and the target proteins of BYF were involved in lipid metabolism, inflammatory response, oxidative stress and focal adhension. In conclusion, BYF exerts long-term therapeutic action on COPD probably through modulating the lipid metabolism, oxidative stress, cell junction and inflammatory response pathways at system level.

Chronic obstructive pulmonary disease (COPD) is an inflammatory disorder characterized by progressive airflow limitation and is thought to result in part from exaggerated pulmonary inflammation in response to chronic aero-pollutant exposure, primarily from smoking^1^. In 2020, COPD is expected to become the fifth leading cause of disease burden and the third leading of mortality, and currently available treatments are largely ineffective^2^. Therefore, novel therapeutic agents are needed urgently for the effective management of COPD.

Traditional Chinese medicinal (TCM) formulae have been extensively used in Eastern Asia for the treatment of COPD^3^. Typically, formulae consist of several types of medicinal herbs, in which one represents the principal component, and others serve as adjuvant ones to assist the effects of the principal component. It is believed that multiple components contained in the formulae could hit multiple targets and exert synergistic and long-term therapeutic efficacies^4^,^5^,^6^. Bufei Yishen formula (BYF), a TCM formula, is composed of twelve medical herbs and has been proven clinically effective for the treatment of COPD^7^. Clinical studies revealed a variety of desirable pharmacological effects of BYF on COPD, such as alleviating the clinical symptoms of stable COPD patients, reducing risk of exacerbation, delaying acute exacerbation, and improving pulmonary function and exercise capacity^7^. In previous study, we successfully constructed a systems pharmacological model by combining active compounds prediction, targets prediction, and network pharmacology, and efficiently identified 216 active compounds from BYF and 195 potential targets mainly related to activation of inflammatory response, immune responses, and matrix metalloproteinases^8^. After treatment with BYF for 12 weeks, we found that BYF exerted short-term therapeutic effect on COPD rats by inhibiting the inflammatory cytokine expression, protease-antiprotease imbalance and the collagen deposition^8^. However, the long-term effect and therapeutic mechanism of BYF on COPD were still unclear. Herein, we conducted a systems-level analysis of the long-term therapeutic effect and underlying mechanism of BYF, which also could provide more system experimental evidences to validate the system pharmacology predictions.

Fortunately, the transcriptomics- proteomics- metabolomics-profiling-technologies integrate the entirety of the biological complement to propose a system-level way of study TCM formula in the form of systems biology^9^,^10^,^11^. Transcriptomic is a promising approach to identify the entire genome^10^. Proteomics could directly study the expressed proteins and protein function in a cellular context^9^. Moreover, metabolomics provides the metabolic information that is the consequence of the transcriptome and proteome^11^,^12^. Furthermore, system pharmacology, as an understanding of the function of the biological system, has been applied to analyze the dynamic interactions between the drugs and biological system by bridging systems biology and pharmacokinetics- pharmacodynamics^13^. Thus, comprehensive analyses of the system pharmacology, transcriptomics, proteomics and metabolomics datasets have the potential to provide a system-wide view of the complex biological processes and the function of the multiple compounds contained in TCM formula.

In present study, COPD rats were treated with BYF from week 9 to 20. We then test the long-term effect of BYF on COPD rats on week 32. We further analyzed the long-term therapeutic mechanism of BYF on COPD rats using the data sets of transcripts, proteins and metabolites derived from the lung tissues. Finally, we integrated system pharmacology, transcriptomics, proteomics and metabolomics data streams to provide a system picture of the long-term mechanisms of BYF in treating COPD rats.

## Results and Discussion

### The long-term effect of BYF on COPD rats

Previous study showed that BYF had been successful applied into the treatment of COPD patients, and exerted short-term therapeutic effect on COPD rats^7^,^8^. In this study, we tested the long-term effect of BYF on COPD rats. A rat model of cigarette smoke- and bacterial infection-induced COPD was established, and treated with BYF from week 9 to 20. The pulmonary functions were assessed from week 0 to 32, and histopathology was analyzed on week 32.

**Figure 1:**
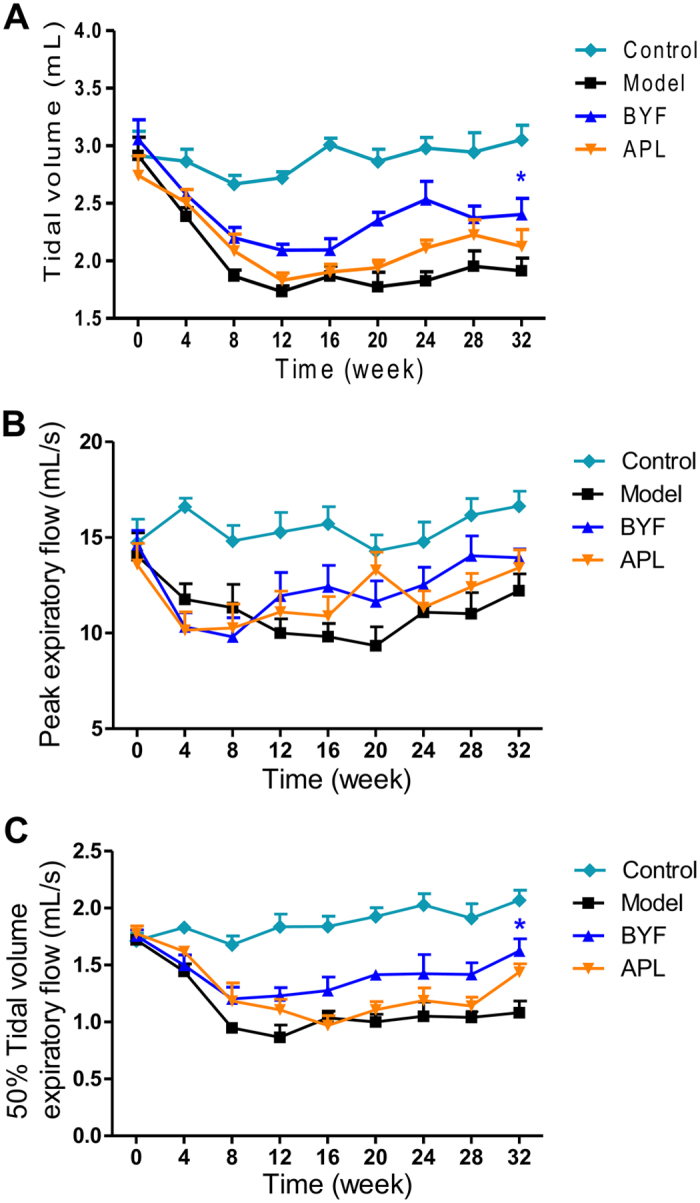
Long-term effect of Bufei Yishen formula (BYF) on the pulmonary function of chronic obstructive pulmonary disease (COPD) rats. The COPD rats were intragastricly treated with 4.44 g/kg of BYF once daily from week 9 to 20. Control was treated with normal saline, and positive control was treated with aminophylline (APL, 2.3 mg/kg) once daily. Tidal volume (TV) (**A**), peak expiratory flow (PEF) (**B**), and 50% tidal volume expiratory flow (EF50) (**C**) were analyzed every fourth week from week 0 to 32. Values represent the means ± SEM. *p < 0.05 vs. model.

**Figure 2:**
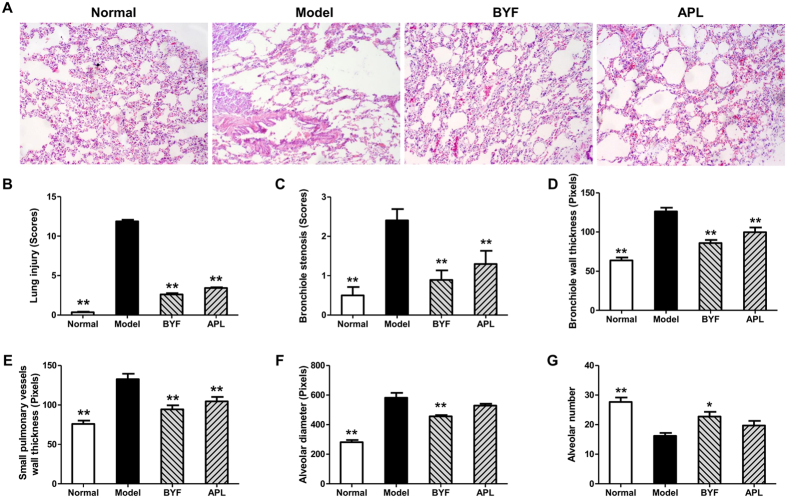
Long-term effect of Bufei Yishen formula (BYF) on the histological changes in lung tissues of chronic obstructive pulmonary disease (COPD) rats. COPD rats were intragastricly treated with BYF (4.44 g/kg) and aminophylline (APL, 2.3 mg/kg) once daily from week 9 to 20. On week 32, the lung tissues of COPD rats were collected. Histological changes were detected using H&E staining (original magnification ×100) (**A**). The lung injury scores of all groups were evaluated (**B**). Bronchial wall thickness (**C**), bronchiole stenosis (**D**), small pulmonary vessels wall thickness (**E**), alveolar number (**F**), and alveolar diameter (**G**) were analyzed. Values represent the means ± SEM. *p < 0.05, **p < 0.01 vs. model.

As shown in Fig. 1, compared with the control rats, the tidal volume (TV), peak expiratory flow (PEF), and 50% tidal volume expiratory flow (EF50) clearly decreased in the model rats from weeks 4 to 32, whereas, this decrease was suppressed by BYF and the classical bronchodilator, aminophylline (APL). In addition, lung injury scores, bronchiole wall thickness, small pulmonary vessels wall thickness, bronchiole stenosis, and alveolar diameter increased in the model rat, which could be significantly suppressed by the treatment of animals with BYF (Fig. 2A–F). Moreover, BYF treatment also markedly increased the alveolar number in COPD rats (Fig. 2G). These data demonstrated that BYF treatment had the long-term therapeutic effect on COPD rats.

### The long-term effect of BYF on inflammatory responses in COPD rats

**Figure 3:**
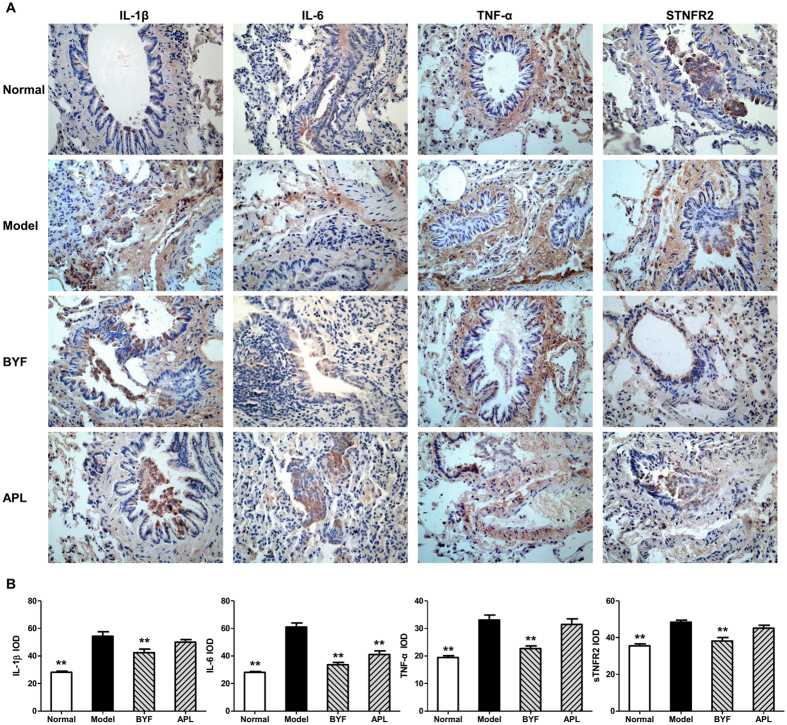
Long-term effect of Bufei Yishen formula (BYF) on the expression of IL-1β, IL-6 TNF-α and sTNFR2, in the lung of chronic obstructive pulmonary disease (COPD) rats. COPD rats were intragastricly treated with BYF (4.44 g/kg) and aminophylline (APL, 2.3 mg/kg) once daily from week 9 to 20. On week 32, the expression of interleukin (IL)-1β, IL-6, tumor necrosis factor (TNF)-α, and soluble TNF-α receptor (sTNFR) 2 in lung tissues were evaluated by immunohistochemistry (magnification, ×100) (**A**). Quantitative analysis for the expression of IL-6, IL-10, TNF-α, and sTNFR2 in the lung of COPD rats (**B**). Values represent the means ± SEM. **p < 0.01 vs. model.

Recent data show that inflammation is associated with morbidity and mortality in patients with COPD^14^,^15^. Previous study showed that BYF treatment could effectively inhibit the inflammatory response in the lungs of COPD rats on week 20^8^. Here, we tested the long-term effect of BYF on the expression of inflammatory mediators in the lung tissues. As shown in Fig. 3, the levels of IL-6, IL-1β, TNF-α, and sTNFR2 increased after the cigarette smoke and bacterial infection exposures, and this increase was significantly inhibited by BYF treatment. These findings demonstrated that BYF treatment could effectively inhibit the inflammatory response in the lung tissues on week 32.

### The long-term effect of BYF on protease-antiprotease imbalance and collagen degradation

**Figure 4:**
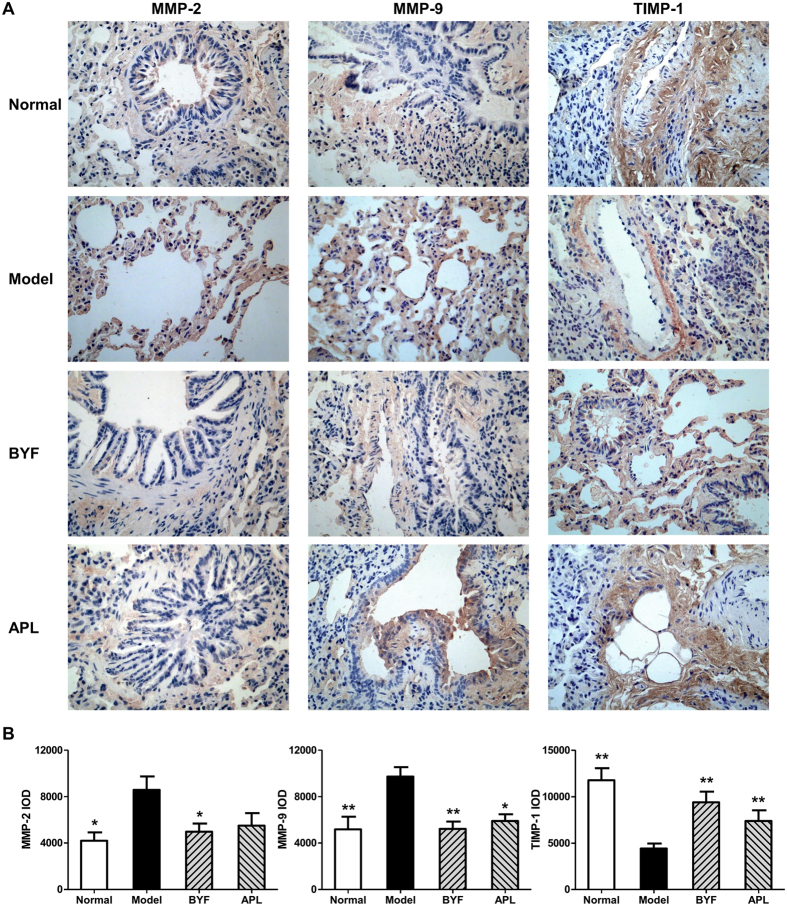
Long-term effect of Bufei Yishen formula (BYF) on the expression of MMP-2, MMP-9 and TIMP-1 in the lung of chronic obstructive pulmonary disease (COPD) rats. COPD rats were intragastricly treated with BYF (4.44 g/kg) and aminophylline (APL, 2.3 mg/kg) once daily from week 9 to 20. On week 32, the expression of matrix metalloproteinase (MMP)-2, MMP-9, and tissue inhibitor of MMP (TIMP)-1 in the lung of COPD rats were evaluated by immunohistochemistry (magnification, ×100) (**A**). Quantitative analysis for the expression of MMP-2, MMP-9, and TIMP-1 in the lung of COPD rats (**B**). Values represent means ± SEM. *p < 0.05, **p < 0.01 vs. model.

**Figure 5:**
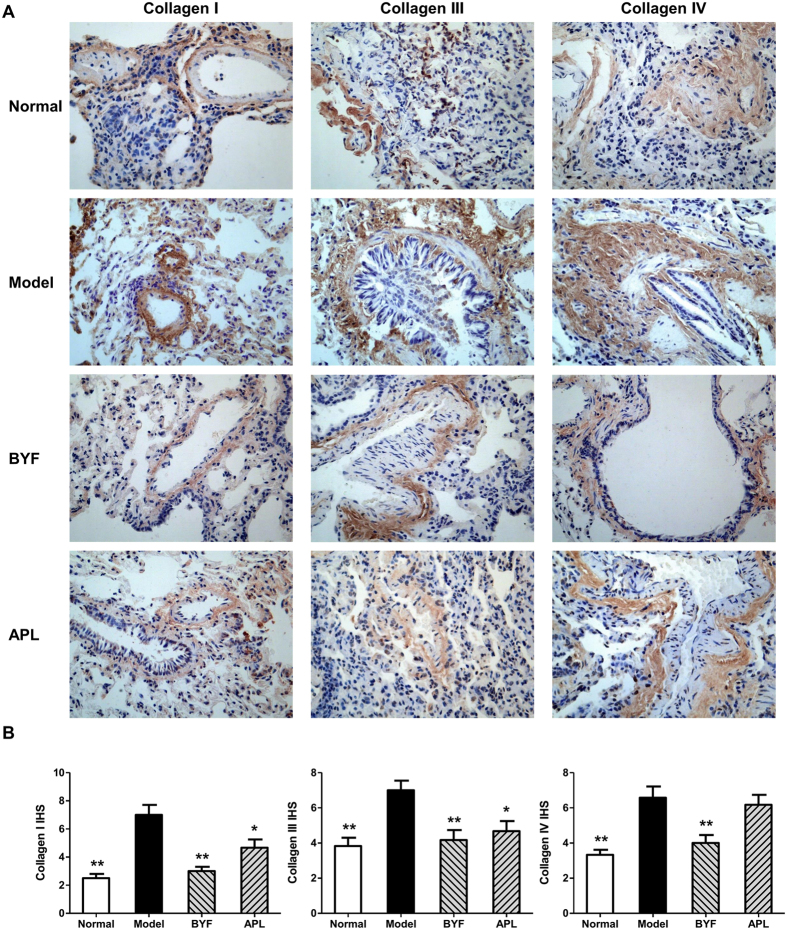
Long-term effect of Bufei Yishen formula (BYF) on the expression of collagens I, III and IV in the lung tissues of chronic obstructive pulmonary disease (COPD) rats. COPD rats were intragastricly treated with BYF (4.44 g/kg) and aminophylline (APL, 2.3 mg/kg) once daily from week 9 to 20. Immunohistochemical analysis for the expression of collagens I, III, and IV in the lung of COPD rats on week 32 (original magnification ×100) (**A**). Quantitative analysis for the expression of collagens I, III, and IV in the lung tissues (**B**). Data are presented as means ± SEM. *p < 0.05, **p < 0.01 vs. model.

The protease-antiprotease imbalance leads to the breakdown of connective tissue components, and induces destruction of the lung parenchyma and development of emphysema, which are the critical mechanisms in the pathogenesis of COPD^16^,^17^. Furthermore, there is increasing interest in the role of MMPs in COPD^18^. Increased concentrations of MMP-2 and MMP-9 were observed in bronchoalveolar lavage fluid from COPD patients, and there is increased activity of MMP-9 and decreased activity of TIMP-1 in the lung parenchyma^19^,^20^. Additionally, the collagen degradation in COPD makes great contribution to the persistent tissue injury and has a role in the airflow obstruction characteristic of this disease^21^. In previous study, we found that BYF could significantly suppress the collagen deposition and protease-antiprotease imbalance in lung tissues on week 20^8^. Therefore, we examined the long-term effect of BYF on the expression of MMP-2, MMP-9, TIMP-1, and collagens I, III, and IV in lung tissues. As shown in Fig. 4, BYF markedly decreased the expression levels of MMP-2 and MMP-9 and increased the level of TIMP-1. In addition, BYF significantly suppressed the expression of collagens I, III, and IV on week 32 (Fig. 5). These results suggested that BYF could inhibit the cigarette smoke- and bacterial infection-induced collagen deposition and protease-antiprotease imbalance by inhibiting the expression of collagens I, III, and IV, MMP-2/9, and increasing the expression of TIMP-1 on week 32.

### Regulation of molecules on the transcriptome, proteome, and metabolome level in lung tissue

The above data showed BYF had long-term therapeutic effect on COPD rats, probably due to its sustained inhibitory effect on the inflammatory cytokine expression, protease-antiprotease imbalance and collagen deposition. Then, to investigate the long-term mechanisms of action of BYF at system level, the transcriptomics-proteomics-metabolomics-profiling-technologies were applied to analyze the molecular aspects of these perturbations in lung tissues.

**Figure 6:**
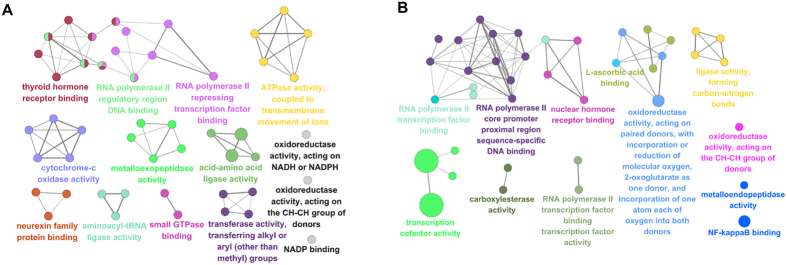
Molecular functions of regulated genes in lung tissues of chronic obstructive pulmonary disease (COPD) rats and Bufei Yishen formula (BYF)-treated rats. The molecular functions of regulated genes in COPD rats (**A**) and regulated genes in BYF-treated rats (**B**) were analyzed by ClueGO: functionally grouped network with terms as nodes linked, and functionally related groups partially overlap; the node size represents the term enrichment significance. The label of only the significant term per group was shown.

Initially we performed a microarray-based RNA expression study on the lung tissues, and about 41000 genes were detected. In these data sets, there were 1063 and 1106 genes differently regulated in COPD model (versus control) and BYF treatment (versus COPD model) rats, respectively (Supplementary Tables S1 and S2). These genes could be attributed to various molecular functions such as oxidoreductase activity, metalloendopeptidase activity, hormone receptor binding, or NF-kappaB binding (Fig. 6A,B). For further analysis, we considered the full data set to extract biological pathway information. As shown in Tables 1 and 2, these transcripts were mapped to many different pathways such as oxidative phosphorylation, focal adhesion or ubiquitin mediated proteolysis pathway.

**Figure 7:**
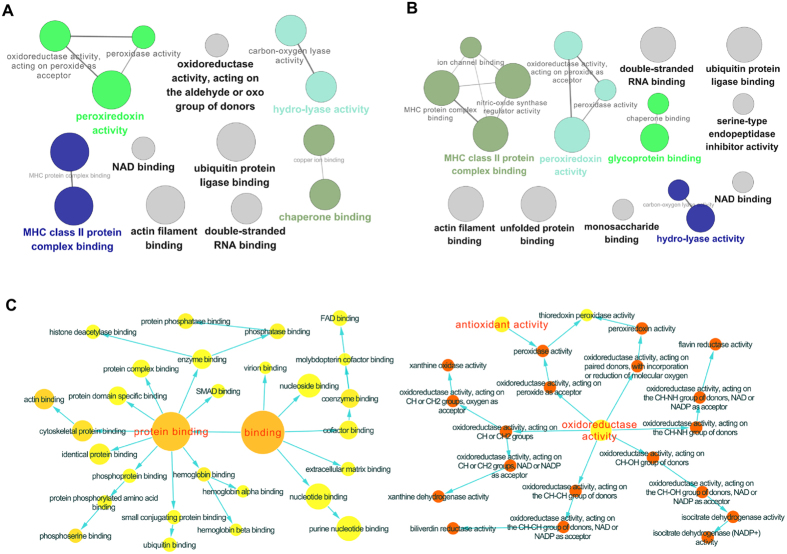
Molecular functions of regulated proteins in lung tissues of chronic obstructive pulmonary disease (COPD) rats and Bufei Yishen formula (BYF)-treated rats. The molecular functions of regulated proteins in COPD rats (**A**) and BYF-treated rats (**B**) were analyzed by ClueGO: functionally grouped network with terms as nodes linked, and functionally related groups partially overlap; the node size represents the term enrichment significance. The predominant function of the overlapping proteins between COPD and BYF-treated rats was analyzed using BiNGO, and the intuitive and customizable visual representation of the results were showed (**C**).

We next analyzed their protein expression profiles using LC-MS based proteomic, and identified 191, 187 proteins differently regulated in COPD model (versus control) and BYF-treated rats (versus COPD model), respectively (Supplementary Tables S3 and S4). As shown in Fig. 7A,B, the molecular functions of these regulated proteins were related to oxidoreductase activity, peroxiredoxin activity, or NAD binding. Furthermore, these regulated proteins were mapped to various pathways such as ECM-receptor interaction, focal adhesion, tight junction, and leukocyte transendothelial migration (Tables 3 and 4).

For further analysis, the COPD model group (191 proteins) shared 132 common proteins of the BYF treated-group (187 proteins). Out of the 132 proteins, expression changes of 70 proteins in COPD model was inhibited by BYF treatment (Supplementary Table S5). The molecular functions of these 70 proteins were mainly related to oxidoreductase activity, antioxidant activity, extracellular matrix binding, and protein phosphatase binding (Fig. 7C), which might be the therapeutic mechanism of action of BYF.

**Figure 8:**
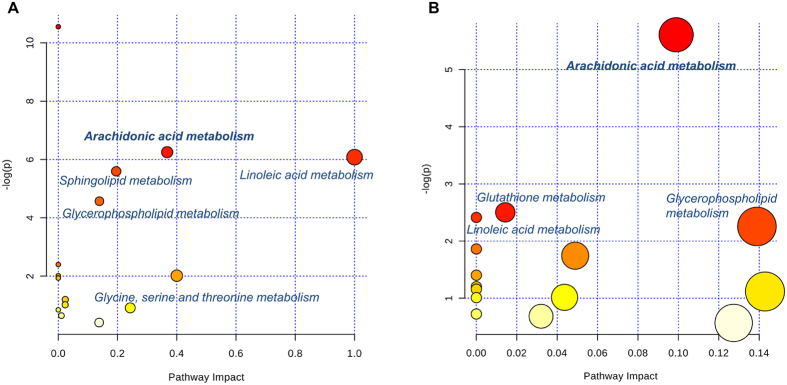
Pathway analysis of the metabolites regulated in lung tissues of chronic obstructive pulmonary disease (COPD) rats and Bufei Yishen formula (BYF)-treated rats. The most relevant pathways were analyzed using the MetaboAnalyst. A Google-map style interactive visualization system was applied to facilitate data exploration and generate pathway views. (**A**) Representative pathway analysis of the metabolites in lung tissues of COPD rats. (**B**) Representative pathway analysis of the metabolites in lung tissues of BYF-treated rats.

Finally, we characterized the metabolic profile of COPD rats and BYF-treated rats using LC-MS based metabolomics. There were 41 and 32 metabolites differently regulated in COPD model (versus control) and BYF treatment (versus COPD model) rats, respectively (Supplementary Tables S6 and S7). For further holistic analysis, we applied MetaboAnalyst to analyze the systematic metabolome changes based on pathway analysis (Tables 5 and 6). The most relevant pathways were analyzed from the perspective of pathway enrichment analysis combined with the topology analysis. As shown in Fig. 8, these metabolites were primarily involved in arachidonic acid, linoleic acid or glutathione metabolism. These metabolic anomalies were found to be primarily involved in lipid metabolism, which are discussed in detail later.

### Holistic Views on genes, proteins and metabolites data

In this above study, we successfully identified a number of regulated genes, proteins and metabolites, which might be involved in the therapeutic effect of BYF on COPD. Thus, to provide the system-wide view of the therapeutic mechanism of BYF in treating COPD, we analyzed the system function of genes, proteins and metabolites by integrating transcriptomics, proteomics, and metabolomics data.

**Figure 9:**
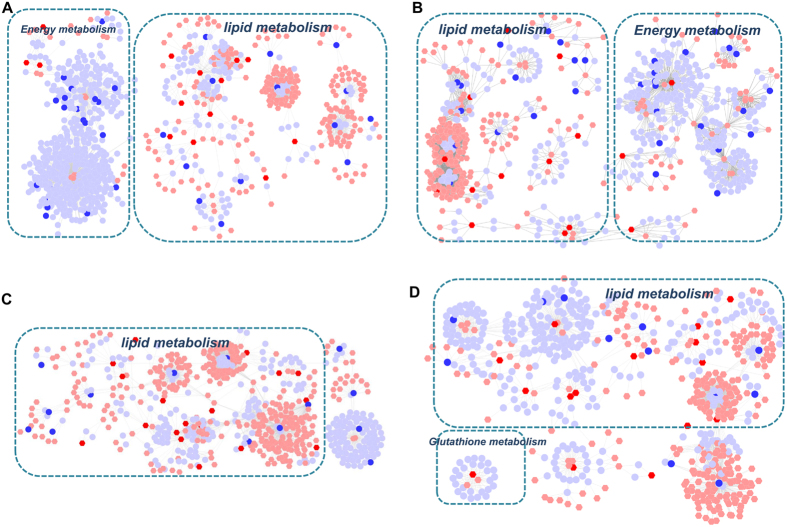
Metabolic correlation networks of the metabolites, genes and proteins regulated in lung tissues of chronic obstructive pulmonary disease (COPD) rats and Bufei Yishen formula (BYF)-treated rats. Using Metscape, the compound reaction network with compounds (hexagons) and metabolic enzymes (rounds) as nodes and reactions as edges was constructed. Inputted genes and proteins were shown in red, inputted compounds were shown in blue. (**A**) Representative metabolites-gene network of COPD model group. (**B**) Representative metabolite-gene network of BYF-treated group. (**C**) Representative metabolites-protein network of COPD model group. (**D**) Representative metabolite-protein network of BYF-treated group.

Initially we applied the Metscape software to test the latent relationships of the metabolite, gene and protein measurements by constructing the correlation network diagram. In Fig. 9A,B, we constructed these two metabolite-gene networks using the tanscriptomics and metabolomics data of COPD and BYF-treated rats. The results showed that these networks were mainly divided into two groups: lipid metabolism and energy metabolism, and most of the metabolites and a number of genes were involved in lipid metabolism. In Fig. 9C,D, based on the metabolite and protein data of COPD and BYF-treated rats, we constructed the metabolite-protein networks. These metabolite-protein networks mainly consisted of lipid metabolism or glutathione metabolism. Interestingly, we also found that more than half of proteins and almost all of metabolites were involved in lipid metabolism.

The prediction from above data is that lipid metabolism disorders were closely related to the occurrence and development of COPD and the long-term therapeutic effect of BYF.

### Integrated analysis of system pharmacology, transcriptomics, proteomics, and metabolomics data

In previous work, we used system pharmacology to identify 195 targets of the BYF (Supplementary Table S8) and investigated the short-term effect of BYF in treating COPD rats^8^. Here, we demonstrated that BYF had long-term effect on COPD rats. Thus, to investigate the system mechanism of this effect of BYF, we combined the transcriptomics, proteomics and metabolomics data streams with system pharmacology targets data to give a system-wide view of the long-term therapeutic mechanism of BYF in treating COPD.

**Figure 10:**
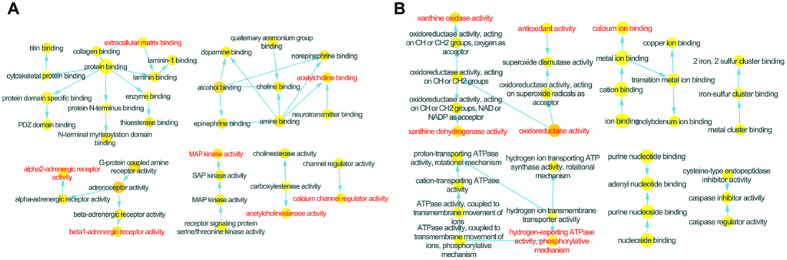
Molecular functions of the overlapping proteins. The predominant function of the proteins was analyzed using BiNGO, and the intuitive and customizable visual representation of the results was showed. The area of a node was proportional to the number of proteins in the test set. (**A**) Representative molecular function of overlapping proteins between the potential targets and transcript measurements in lung tissues of Bufei Yishen (BYF)-treated rats. (**B**) Representative molecular function of overlapping proteins between the potential targets and proteome measurements in lung tissues of BYF-treated rats.

**Figure 11:**
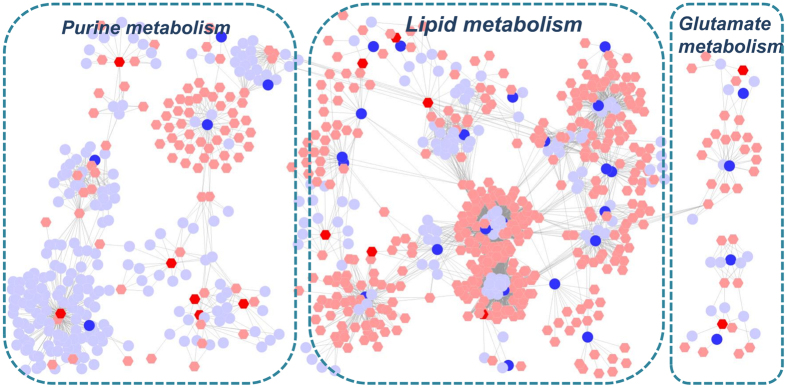
Metabolic correlation networks of the metabolites regulated in Bufei Yishen formula (BYF)-treated rats and target proteins. Using Metscape, the compound reaction network with compounds (hexagons) and metabolic enzymes (rounds) as nodes and reactions as edges was constructed. Inputted compounds were shown in blue, inputted target proteins were shown in red.

Firstly, there were 8 overlapping proteins (katA, MAPK14, ACHE, ADRB1, CALM1, ADRA2B, ampC, gyrB) between the potential targets of BYF and transcript measurements of BYF-treated rats. These 8 overlapping proteins could be attributed to various molecular functions such as MAP kinase activity, adrenergic receptor activity, acetylcholinesterase activity, acetylcholine binding and extracellular matrix binding (Fig. 10A). Furthermore, we identified 9 overlapping proteins (ampC, ATP5B, CALM1, COL1A2, gyrB, HSPA5, katA, SOD1 XDH) between the targets of BYF and proteins regulated in BYF-treated rats. For further functional analysis, the molecular functions of these 9 proteins were mainly related with oxidoreductase activity, antioxidant activity, and xanthine dehydrogenase/oxidase activity (Fig. 10B). Finally, using Metscape software, we analyzed the latent relationships of the target proteins and metabolites regulated in BYF-treated rats by constructing the correlation network. As shown in Fig. 11, we found that the metabolite-target protein network mainly consisted of lipid metabolism.

**Figure 12:**
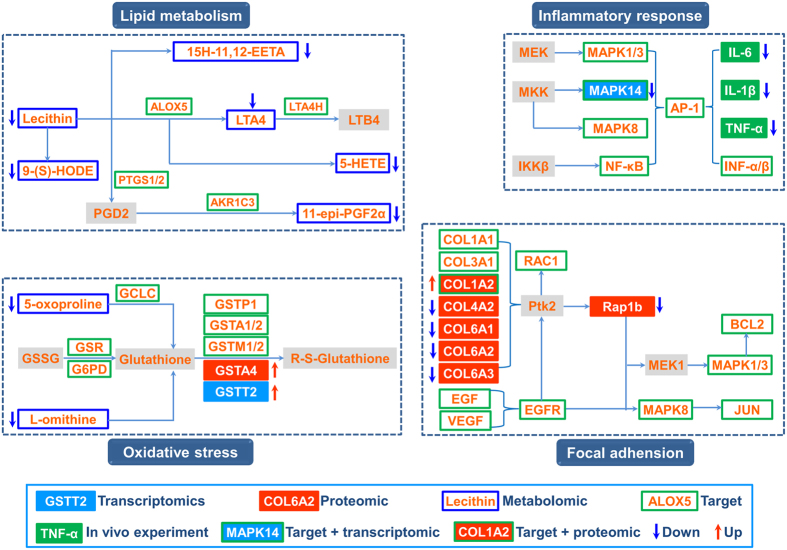
Integrated analysis of the potential targets of Bufei Yishen formula (BYF), transcriptomics, proteomics, and metabolomics regulations in lung tissues of BYF-treated rats. The potential targets, transcriptomics, proteomics and metabolomics data were shown as rectangles with different colors. Red arrow stands for up regulated, blue arrow for down regulated, and gray rectangle for unregulated.

Based on above comprehensive analysis, we then provided a system-wide view of the therapeutic mechanisms of long-term effect of BYF. In Fig. 12, the system-level picture was divided into four groups: lipid metabolism, inflammatory response, oxidative stress and focal adhension pathway.

As discussed above, arachidonic acid metabolism and linoleic acid metabolism were the significant deregulated pathways in lung tissues of BYF-treated rats. The levels of metabolites such as Lecithin, 9-(S)-HODE 15H-11, 12-EETA, LTA4, 5-HETE, 11-epi-PGF2α were suppressed by BYF treatment. In these metabolism pathways, many metabolites such as LTA4, PGF2, LTB4 participated in inflammatory processes in the airways of patients with COPD. Furthermore, in the system pharmacology predictions, the important metabolic enzymes such as ALOX5, PTGS1/2, LTA4H, and AKR1C3 were the potential targets of BYF, which involved in these deregulated pathways. In system pharmacology and *in vivo* experimental study, we showed that MAPK1/3 (ERK1/2), MAPK14 (p38), MAPK8 (JNK) and NF-κB were the potential targets of BYF, and inflammatory cytokines, including IL-1β, IL-6 and TNF-α were suppressed by BYF treatment. These data indicated that BYF could effectively suppress inflammatory responses by regulating lipid metabolism, the pre-inflammatory cytokines production as well as their corresponding pathways activation.

The oxidative stress can trigger sustained inflammatory responses. Moreover, the common characteristic of COPD is that it suffers from oxidative stress^22^,^23^. Glutathione is one of the main antioxidants, which can protect cells from oxidative damage by decreasing the level of reactive oxygen species^24^,^25^. In this work, we detected many metabolites (5-oxoproline, L-omithine), tanscripts (GSTA4), proteins (GSTT2) and the potential targets (GCLC, GSR, G6PD, GSTP1, GSTA1/2, GSTM1/2), which were involved in glutathione metabolism. In addition the antioxidant proteins such as SOD1 (superoxide dismutase), involved in the pathogenesis of COPD, was up-regulated by BYF treatment (Supplementary Table S4). These results suggested that BYF achieved its anti-inflammatory activity probably by regulating glutathione metabolism and increasing the level of antioxidants.

Moreover, focal adhension was the significant deregulated pathways in proteomic measurements of BYF-treated rats and the potential targets of BYF (Tables 4 and 7). Specially, the activation of MAPK1/3, 8, and JUN, the potential targets of BYF, is to up-regulate the transcript levels of pre-inflammatory cytokines^26^,^27^,^28^. Taken together, these results demonstrated that BYF had long-term effect on COPD by regulating multiple biological functions, such as lipid metabolism, inflammatory response, oxidative stress and focal adhension pathway.

Integrating omic approaches is becoming more important in pathological study and will most likely change the way we approach TCM biological investigations. However, attempts thus far to combine omic data streams have been met with limited success^29^. In the present study, we have brought together experts from the various fields of *in vivo* experiment, transcriptomic, proteomic, metabolomic and system pharmacology modeling. The major aim was to investigate the benefit of integrating omic data streams and system pharmacology for application in elucidating system mechanisms of BYF. However, it is an undeniable fact that the approach of integrated omics analysis has many flaws. When we seek for common data among the system pharmacology and 3-omics data, we may be inclined to find enrichment in the desired gene function categories. In our subsequent work, we would attempted to construct system-wide methods to analyze the overall consistency among the 3-omics data and system pharmacology data.

## Conclusion

In this work, we proposed a system-level approach to investigate the long-term effect and its therapeutic mechanism of BYF on COPD rats by systematically incorporating system pharmacology, transcriptomics, proteomics and metabolomics data analysis. We treated COPD rats with BYF from week 9 to 20, and observed that BYF exerted long-term therapeutic effect on COPD rats and suppressed the inflammatory cytokine expression, protease-antiprotease imbalance and the collagen deposition on week 32. To dissect the mechanism of its long-term therapeutic effect at system level, we characterized the transcriptomics, proteomics and metabolomics profiles of COPD and BYF-treated COPD rats. The regulated transcripts, proteins and metabolites were attributed to multiple functions, such as oxidoreductase activity, antioxidant activity and lipid metabolism. Finally, we conducted a systems-level analysis by integrating system pharmacology, transcriptome, proteome, and metabolome data. The system-level results indicated that BYF achieved its long-term ameliorative effect over COPD probably by regulating lipid metabolism, inflammatory response, oxidative stress and focal adhension pathways at system level.

In summary, integrative application of system pharmacology and 3-omics technologies have potential implications toward understanding the system-level mechanism of long-term action of traditional Chinese medicine, which may propel the new ways toward exploring new drug therapies for complex diseases.

## Materials and Methods

### Chemicals and animals

Aminophylline was obtained from Shandong Xinhua Pharmaceutical Co., LTD. (Shandong, China). *Klebsiella pneumoniae* (strain ID: 46114) was purchased from the National Center for Medical Culture Collection (CMCC, Beijing, China). The tobacco (Filter tip cigarette; tobacco type, tar: 10 mg; nicotine content: 1.0 mg; carbon monoxide: 12 mg) was obtained from Henan Tobacco Industry (Zhengzhou, China). Antibodies against interleukin (IL)-1β, IL-6, tumor necrosis factor (TNF)-α, soluble TNF-α receptor 2 (sTNFR2), collagen I, collagen III, collagen IV, matrix metalloproteinase (MMP)-2, MMP-9, and tissue inhibitor of MMP (TIMP)-1 were purchased from Santa Cruz Biotechnology (Santa Cruz, CA, USA). Mayer’s hematoxylin and 1% eosin alcohol solution were purchased from MUTO Pure Chemicals (Tokyo, Japan). Forty-two Sprague-Dawley rats (21 male and 21 female; 200 ± 20 g) were purchased from the Experimental Animal Center of Henan Province (Zhengzhou, China). The animals were housed in cages with free access to food and tap water under standard conditions of humidity (50 ± 10%), temperature (25 ± 2 °C), and light (12 h light/12 h dark cycle). The animal experiments were conducted with the approval of the Experimental Animal Care and Ethics Committee of the First Affiliated Hospital, Henan University of Traditional Chinese Medicine. The methods were carried out in accordance with the approved guidelines of the Experimental Animal Care and Ethics Committee of the First Affiliated Hospital, Henan University of Traditional Chinese Medicine.

### COPD model and drug administration

COPD rat model and BYF formula was prepared as described in the previous study^30^. Briefly, thirty-two rats were placed into a closed box exposed to the tobacco and repeated *Klebsiella pneumoniae* infections^30^. The components of BYF were as follows: Ginseng Radix et Rhizoma 9 g, Astragali Radix 15 g, Corni Fructus 12 g, Lycii Fructus 12 g, Schisandrae Chinensis Fructus 9 g, Epimedii Herba 9 g, Fritillariae Thunbergii Bulbus 9 g, Paeoniae Rubra Radix 9 g, Pheretima 12 g, Perillae Fructus 9 g, Ardisiae Japonicae Herba 15 g, Citri Reticulatae Pericarpium 9 g. The herbal drugs were identified and prepared in fluid extract^8^.

On week 9, thirty COPD rats were randomly divided into three groups. Then COPD rats were intragastrically administrated with normal saline (2 mL), BYF (4.44 g/kg, 0.5 g/ml), and aminophylline (2.3 mg/kg) every day during week 9 to 20. The control rats also were intragastrically treated with normal saline (2 mL) for the same amount of time. On week 32, all rats were sacrificed, and the heart and lung tissues were collected. The experiments were conducted in accordance with guidelines of the Committee on the Care and Use of Laboratory Animals of the First Affiliated Hospital, Henan University of Traditional Chinese Medicine, China.

### Respiratory data collection

Respiratory data were analyzed using unrestrained pulmonary function testing plethysmography (Buxco Inc., Wilmington, NC, USA) conducted every fourth week from weeks 0 to 32. Rats were placed inside a closed unrestrained Whole Body Plethysmograph (UWBP, Buxco Electronics, Troy, NY, U.S.A.) connected to a transducer and computer. Each chamber was calibrated to its respective transducer. We focused on three measures: tidal volume (TV), peak expiratory flow (PEF), and 50% tidal volume expiratory flow (EF50).

### Histological analyses

Lung tissue samples were fixed in 10% formalin, paraffin-embedded and then cut into 4-μm sections. For histological examination, sections were stained with Mayer’s hematoxylin and then with 1% eosin alcohol solution (H&E staining). The morphological changes were examined using an Olympus BX51 microscope (Tokyo, Japan).

Bronchia, lung injury, and bronchiole stenosis were examined under an optical microscope. Alveolar number, alveolar diameter, small pulmonary vessels, and bronchial wall thickness were analyzed using Image-Pro Plus (IPP) 6.0 software (Media Cybernetics, MD, USA). The morphometric analysis at the light microscopic level was performed by an investigator blinded to the study protocol.

Immunohistochemistry (IHC) was used to analyzed MMP-2, MMP-9, TIMP-1, IL-6, IL-1β, TNF-α, sTNFR2 and collagen I, III, IV expression. Briefly, lung tissue paraffin sections (4 μm) were heat fixed, deparaffinized and rehydrated through graded alcohols to distilled water. Then, the sections were subjected to antigen retrieval and treated with 3% hydrogen peroxide to block endogenous peroxidase activity. The primary antibodies were diluted by 1:100. The sections were incubated with the primary antibodies (Santa Cruz Biotechnology) against MMP-2, MMP-9, TIMP-1, IL-6, IL-1β, TNF-α, sTNFR2, and collagens I, III, and IV overnight. Then, sections were incubated with the secondary antibody for 2 h, and chromogen substrate reagent DAB were used to detect the antigen. A brown color reaction with distinct morphology was developed with DAB in the peroxidase system. Then, sections were counter-stained with hematoxylin. Finally, samples were inspected and measured using IPP 6.0 software (Media Cybernetics, MD, USA).

### Gene expression analyses

Total RNA of the lung tissues was isolated using Trizol reagent (Invitrogen, Breda, Netherlands) and purified using a Qiagen RNeasy Micro kit (Qiagen, Venlo, Netherlands). RNA integrity was analyzed by standard agarose gel electrophoresis and ethidium bromide staining. Then, RNA was PCR amplified by First Strand cDNA Synthesis Kit (Roche, Basel, Switzerland), labeled using an Agilent Quick Amp kit (Agilent Technologies, Santa Clara, CA, USA) and hybridized with Agilent Whole Rat Genome Oligo Microarray (4 × 44 K). Finally, the slides were washing and analyzed using an Agilent DNA microarray scanner (part number G2505B). The data were analyzed using Agilent GeneSpring GX software version 11.0, were subsequently filtered for significant detection (Student’s t-test screening, p < 0.05) and differential expression *vs.* COPD model rats (fold change, |log ratio| > 1). Shanghai Biotechnology Corporation provided technical support for detecting gene expression.

### Protein expression analysis

Proteins were isolated from lung tissue from each of the three experimental groups. Briefly, the lung tissues were lysed using the lysis buffer (4% SDS, 0.1 M DTT, 0.1 M Tris pH 8.0) and homogenized in a mechanical homogenizer (Retsch Technology, Haan, Germany). The lysates were clarified by centrifugation and stored at −80 °C. For proteolytic digestion, trypsin (Roche, Mannheim, Germany) solution were added to the protein solution and incubated for 24 h at 37 °C. Then, each of the samples was reconstituted with isopropanol individually. Tryptic peptides were labeled with 8-plex iTRAQ labeling reagents (ABSCIEX, Darmstadt, Germany) according to the manufacturer’s protocol.

For strong cation exchange fractionation, the buffers A (10 mM KH2PO4 in 25% ACN at pH 3) and B (10 mM KH2PO4 and 2 M KCl in 25% ACN at pH 3) were used as the mobile phase. The peptide mixtures were dissolved in buffer A and then loaded onto a PolySULFOETHYL A column. Peptides were fractionated using buffer B. Fractions were collected, dried and dissolved in 0.1% FA and used for LC-MS analysis.

LC-MS/MS analysis was performed using a Nano liquid chromatography (daojing, Japan) coupled on-line to a Q-TOF mass spectrometer (Bruck). Water with 0.1% FA and ACN with 0.1% FA were used as the mobile phase. The peptide samples were added into a pulled tip column (15 cm × 100 μm id) containing C18 Reprosil, 5 μm particles (Nikkyo Technos, Tokyo, Japan) and then separated.

The reporter ion ratio for each identified peptide was analyzed by Mascot. The proteomics data were analyzed by the loess and global median normalization, and then log2-transformed. Statistical significance was tested by one sample t test. p values < 0.05 were considered as statistically significant. Fold change higher than 1.0 for up-regulation or lower than 1.0 for down-regulation.

### Metabolites analysis

The lung tissue was mixed with cold methanol/water (4:1, v:v) and then homogenized using a highspeed blender. After ultrasonication, the sample was placed on ice for 20 minutes and then deproteinized by centrifugation. Finally, the supernatant was freeze-dried and dissolved in methanol/water (4:1, v:v).

Metabolic profiling analysis was performed using an Agilent-1200 LC system coupled to an Agilent-6520 Q-TOF mass spectrometry. Chromatographic separation was conducted on an Eclipse plus C18 column (1.8 μm, 3.0 × 100 mm2, Agilent). The flow rate was 0.3 mL/min and the mobile phase was ultrapure water with 0.1% formic acid (A) and acetonitrile with 0.1% formic acid (B). The sample injection volume was 5 μL. The parameters of mass detection were set as followed: drying gas (N2) flow rate, 10 L/min; gas temperature, 330 °C; the nebulizer gas pressure, 40 psig; capillary voltage was 4000 V in positive mode and 3000 V in negative mode; fragmentor, 135 V; skimmer, 65 V; scan range was from m/z 100 to 1000^31^.

The LC-MS raw MS data were exported using Agilent Mass Hunter Qualitative Analysis Software (Agilent Technologies, Palo Alto, CA, USA). The data were normalized to the total area. The total integrated area of each sample was normalized to 1,000. Metabolite profile analysis were performed using partial least-squares discriminant analysis (PLS-DA) in software SIMCA-P (Ver 11.0, Umetric, Umea, Sweden)^32^. Significance was determined using the Student’s t-test and the one-way analysis of variance on the mean of three different experiments. p values < 0.05 were considered as statistically significant.

### Gene, Protein and metabolite set enrichment, network, and pathway analyses

The molecular function of transcripts and proteins were analyzed using BINGO, a Cytoscape v3.1.1 plugin^33^. Pathway enrichment analysis of transcripts and proteins were performed using the DAVID and KEGG database. We considered regulated pathways only as statistically significant, if the p was < 0.05. Metscape was applied to analyze the integrated pathway of gene, protein and metabolomics data^34^. We also applied ClueGO, a Cytoscape plugin, to explore the molecular function of the genes and proteins^35^. In the MetaboAnalyst 3.0 was applied to identify the most relevant pathways of the metabolites^36^.

### Statistical analysis

Differences between groups were determined by one-way analysis of variance (ANOVA) with the SPSS 19.0 software package (SPSS, Chicago, IL, USA). Values are expressed as means ± SEM. For all tests, a two-sided p value less than 0.05 was considered significant.

## Additional Information

**How to cite this article**: Li, J. *et al.* System biology analysis of long-term effect and mechanism of Bufei Yishen on COPD revealed by system pharmacology and 3-omics profiling. *Sci. Rep.* **6**, 25492; doi: 10.1038/srep25492 (2016).

## Figures and Tables

**Table 1:** Analyzed pathways of transcriptomics data differently regulated in lung tissue of chronic obstructive pulmonary disease rats.

Term	Count	%	PValue
Ribosome	16	0.2530	0.0000
Ubiquitin mediated proteolysis	12	0.1898	0.0008
Neurotrophin signaling pathway	11	0.1740	0.0027
Spliceosome	10	0.1582	0.0067
Long-term potentiation	7	0.1107	0.0108
Oxidative phosphorylation	10	0.1582	0.0145
Non-small cell lung cancer	6	0.0949	0.0147
Renal cell carcinoma	6	0.0949	0.0437
Parkinson’s disease	9	0.1423	0.0457
Focal adhesion	11	0.1740	0.0469
GnRH signaling pathway	7	0.1107	0.0480
Alzheimer’s disease	11	0.1740	0.0497

**Table 2:** Analyzed pathways of transcriptomics data differently regulated in lung tissue of Bufei Yishen formula-treated rats.

Term	Count	%	PValue
Huntington’s disease	14	0.2391	0.0069
Oxidative phosphorylation	10	0.1708	0.0291
Lysosome	9	0.1537	0.0314
Parkinson’s disease	10	0.1708	0.0355
Alzheimer’s disease	12	0.2049	0.0472
Leukocyte transendothelial migration	8	0.1366	0.0484

**Table 3:** Analyzed pathways of proteomics data differently regulated in lung tissue of chronic obstructive pulmonary disease rats.

Term	Count	%	PValue
ECM-receptor interaction	8	0.3666	0.0002
Focal adhesion	11	0.5041	0.0006
Leukocyte transendothelial migration	7	0.3208	0.0069
Glycolysis/Gluconeogenesis	6	0.2750	0.0079
Propanoate metabolism	4	0.1833	0.0125
Pyruvate metabolism	4	0.1833	0.0196
Tryptophan metabolism	4	0.1833	0.0254
Valine, leucine and isoleucine degradation	4	0.1833	0.0303
Small cell lung cancer	5	0.2291	0.0343
Regulation of actin cytoskeleton	8	0.3666	0.0348
Tight junction	6	0.2750	0.0433
Nitrogen metabolism	3	0.1375	0.0450
Pentose phosphate pathway	3	0.1375	0.0487

**Table 4:** Analyzed pathways of proteomics data differently regulated in lung tissue of Bufei Yishen formula-treated rats.

Term	Count	%	PValue
Focal adhesion	14	0.6591	0.0000
ECM-receptor interaction	8	0.3766	0.0002
Regulation of actin cytoskeleton	11	0.5179	0.0013
Leukocyte transendothelial migration	8	0.3766	0.0018
Pentose phosphate pathway	4	0.1883	0.0056
Neurotrophin signaling pathway	7	0.3296	0.0126
Tight junction	7	0.3296	0.0145
Glycolysis/Gluconeogenesis	5	0.2354	0.0398
Viral myocarditis	5	0.2354	0.0443
Antigen processing and presentation	5	0.2354	0.0459

**Table 5:** Analyzed pathways of metabolomics data differently regulated in lung tissue of chronic obstructive pulmonary disease rats.

Term	Total	Expected	Hits	Raw p
Biosynthesis of unsaturated fatty acids	42	0.65906	6	2.62E-05
Arachidonic acid metabolism	36	0.56491	4	0.0019285
Linoleic acid metabolism	5	0.078459	2	0.0022856
Sphingolipid metabolism	21	0.32953	3	0.0037176
Glycerophospholipid metabolism	30	0.47076	3	0.010353
Cyanoamino acid metabolism	6	0.094151	1	0.09069
alpha-Linolenic acid metabolism	9	0.14123	1	0.13304
Methane metabolism	9	0.14123	1	0.13304
Fatty acid biosynthesis	43	0.67475	2	0.1443
Steroid hormone biosynthesis	70	1.0984	2	0.3014
Cysteine and methionine metabolism	28	0.43937	1	0.36059
Glycine, serine and threonine metabolism	32	0.50214	1	0.40061
Steroid biosynthesis	35	0.54922	1	0.42905
Primary bile acid biosynthesis	46	0.72183	1	0.52269
Aminoacyl-tRNA biosynthesis	67	1.0514	1	0.66232

**Table 6:** Analyzed pathways of metabolomics data differently regulated in lung tissue of Bufei Yishen formula-treated rats.

Term	Total	Expected	Hits	Raw p
Arachidonic acid metabolism	36	0.66762	4	0.0036618
Glutathione metabolism	26	0.48217	2	0.081872
Linoleic acid metabolism	5	0.092725	1	0.089472
Glycerophospholipid metabolism	30	0.55635	2	0.10478
alpha-Linolenic acid metabolism	9	0.1669	1	0.15546
Vitamin B6 metabolism	9	0.1669	1	0.15546
Pyrimidine metabolism	41	0.76034	2	0.1748
Pantothenate and CoA biosynthesis	15	0.27817	1	0.24589
beta-Alanine metabolism	19	0.35235	1	0.30092
Butanoate metabolism	20	0.3709	1	0.31406
Sphingolipid metabolism	21	0.38944	1	0.32697
Purine metabolism	68	1.2611	2	0.36233
Alanine, aspartate and glutamate metabolism	24	0.44508	1	0.36429
Steroid biosynthesis	35	0.64907	1	0.48486
Amino sugar and nucleotide sugar metabolism	37	0.68616	1	0.50428
Arginine and proline metabolism	44	0.81598	1	0.56685

**Table 7:** Analyzed pathways of the potential target of Bufei Yishen formula.

Term	Count	%	PValue
Neuroactive ligand-receptor interaction	30	0.7413	0.0000
Amyotrophic lateral sclerosis (ALS)	12	0.2965	0.0000
Pathways in cancer	27	0.6672	0.0000
Drug metabolism	12	0.2965	0.0000
Calcium signaling pathway	19	0.4695	0.0000
Bladder cancer	10	0.2471	0.0000
Metabolism of xenobiotics by cytochrome P450	11	0.2718	0.0000
Non-small cell lung cancer	10	0.2471	0.0000
Glutathione metabolism	9	0.2224	0.0001
Colorectal cancer	11	0.2718	0.0001
Small cell lung cancer	11	0.2718	0.0001
Pancreatic cancer	10	0.2471	0.0001
Prostate cancer	11	0.2718	0.0001
VEGF signaling pathway	10	0.2471	0.0002
Gap junction	10	0.2471	0.0007
Thyroid cancer	6	0.1483	0.0010
Focal adhesion	15	0.3706	0.0011
GnRH signaling pathway	10	0.2471	0.0013
Glioma	8	0.1977	0.0015
Alzheimer’s disease	13	0.3212	0.0015
Progesterone-mediated oocyte maturation	9	0.2224	0.0022
Prion diseases	6	0.1483	0.0024
T cell receptor signaling pathway	10	0.2471	0.0026
Arginine and proline metabolism	7	0.1730	0.0030
Melanoma	8	0.1977	0.0030
Fc epsilon RI signaling pathway	8	0.1977	0.0051
Toll-like receptor signaling pathway	9	0.2224	0.0061
Neurotrophin signaling pathway	10	0.2471	0.0065
NOD-like receptor signaling pathway	7	0.1730	0.0065
Apoptosis	8	0.1977	0.0093
Adipocytokine signaling pathway	7	0.1730	0.0095
Vascular smooth muscle contraction	9	0.2224	0.0111
Insulin signaling pathway	10	0.2471	0.0111
Renal cell carcinoma	7	0.1730	0.0117
Endometrial cancer	6	0.1483	0.0132
MAPK signaling pathway	15	0.3706	0.0137
Caffeine metabolism	3	0.0741	0.0142
B cell receptor signaling pathway	7	0.1730	0.0161
Arachidonic acid metabolism	6	0.1483	0.0178
Graft-versus-host disease	5	0.1235	0.0210
Phenylalanine metabolism	4	0.0988	0.0210
Tryptophan metabolism	5	0.1235	0.0228
Oocyte meiosis	8	0.1977	0.0302
ErbB signaling pathway	7	0.1730	0.0310
Tyrosine metabolism	5	0.1235	0.0312
p53 signaling pathway	6	0.1483	0.0374
Epithelial cell signaling in Helicobacter pylori infection	6	0.1483	0.0374
Type II diabetes mellitus	5	0.1235	0.0385
Complement and coagulation cascades	6	0.1483	0.0395
